# Combining results from hip impingement and range of motion tests can increase diagnostic accuracy in patients with FAI syndrome

**DOI:** 10.1007/s00167-020-06005-5

**Published:** 2020-04-25

**Authors:** Anders Pålsson, Ioannis Kostogiannis, Eva Ageberg

**Affiliations:** 1 Department of Health Sciences, Lund University, PO Box 157, 22100 Lund, Sweden; 2 Division of Orthopaedics, Department of Clinical Sciences, Lund University, Lund, Sweden

**Keywords:** Hip, Groin, Femoracetabular impingement, Physical examination, Reliability, Diagnosis

## Abstract

**Purpose:**

Clinical examination is an important part in the diagnosis of femoroacetabular impingement (FAI) syndrome. However, knowledge on reliability and validity of clinical diagnostic tests is scarce. The aims were to evaluate the inter-rater agreement and diagnostic accuracy of clinical tests to detect patients with FAI syndrome.

**Methods:**

Eighty-one patients (49% women) were recruited. Two experienced raters performed impingement and range of motion (ROM) tests. Three criteria had to be fulfilled for the diagnosis of FAI syndrome: (1) symptoms; (2) CAM and/or Pincer morphology; and (3) being responder to intra-articular block injection. For inter-rater agreement, the Cohen’s kappa statistics were used (0.41–0.60 = moderate, 0.61–0.80 = substantial agreement). For diagnostic accuracy, sensitivity, specificity, positive and negative predictive values were calculated.

**Results:**

Anterior impingement test (AIMT), FADIR test and FABER test showed kappa values above 0.6. All passive hip ROM, except extension, had kappa values above 0.4. AIMT and FADIR showed the highest sensitivity, i.e., 80%, with a specificity of 26% and 25%, respectively. Passive hip ROM in internal rotation with neutral hip position had a sensitivity of 29% and a specificity of 94%.

**Conclusion:**

The AIMT, FADIR and FABER tests were reliable between two experienced raters, while results from different raters for hip ROM should be interpreted with caution. The AIMT and FADIR test can only be used to rule out patients with FAI syndrome, while evaluation of ROM in internal rotation with neutral position may be more suitable to rule in patients with FAI syndrome.

**Level of evidence:**

II.

## Introduction

Long-standing hip and groin pain is common among physically active people participating in high-impact sports [22, 47, 53] and among less physically active people [29, 41]. Long-standing hip and groin pain often limits a person’s ability to participate in physical as well as daily activities and reduces his or her quality of life [49, 54].

Diagnostics are challenging to perform in patients with long-standing hip and groin pain due to the likely multi-structural origin of the pain, where both intra- and extra-articular pathologies may coexist [27, 46]. A consensus statement on the terminology and definitions for describing symptoms presented in the hip/groin area was recently published [52]. The diagnostic classification system includes the following subgroups: (1) groin pain, including adductor-related, iliopsoas-related, inguinal-related, and pubic-related groin pain; (2) hip-related groin pain; and (3) other types of groin pain [52]. The most common causes of hip-related groin pain appear to be femoroacetabular impingement (FAI) syndrome and labral tears [10].

A triad of symptoms, clinical signs and radiological findings should be used to diagnose FAI syndrome [25]. The symptoms of FAI syndrome include motion-related or position-related pain in the hip or groin, with or without symptoms such as clicking, catching, locking and stiffness. The clinical signs include the reproduction of the patient’s typical pain during hip impingement tests and limited hip range of motion (ROM). Finally, the radiological findings include an oval shape of the femoral head (CAM morphology) or overcoverage of the femoral head by the acetabulum (pincer morphology). To further confirm the diagnosis, image-guided intra-articular block injections can be used when all the other criteria have been met [11, 25].

The treatment of FAI syndrome involves education, a modification of the patient’s activity level, and exercise-based therapy [13, 25]. A subgroup of patients may benefit from a combination of exercise-based therapy and hip surgery, at least in the short term [26]. Due to advances in hip arthroscopy, the number of surgical procedures that have been performed has increased dramatically over the last decade [17, 18]. The diagnostic challenge in patients with hip and groin pain, as well as the increasing number of hip arthroscopy procedures, may lead to a higher number of patients referred to tertiary care for consideration for surgery. In a recent study, we showed that only 50% of those who were referred to tertiary care were categorized as having hip-related groin pain [40]. To avoid unnecessary referral to tertiary care and potentially avoid unnecessary invasive examinations, including those involving radiographs, reliable and valid clinical tests should be performed in clinical examinations.

To the best of our knowledge, the evaluation of the hip ROM as an additional diagnostic test for FAI syndrome has not been studied. Additionally, previous studies on the accuracy of clinical tests in diagnosing FAI syndrome have mainly used the visualization of FAI morphology as a reference standard only [43]. The use of a combination of symptoms, radiological findings, and outcomes of intra-articular block injection may be more appropriate. We hypothesized that using both impingement tests and an evaluation of hip ROM as well as a more comprehensive reference standard (symptoms, radiological findings, intra-articular block injection) would lead to improved diagnostic accuracy of FAI syndrome.

The aims of the present study were to (1) evaluate the inter-rater agreement of the clinical assessment of the hip and (2) evaluate the diagnostic accuracy of the clinical examination, including hip impingement and passive hip ROM tests, in detecting patients with symptoms of and radiologically verified hip morphology corresponding to FAI syndrome (CAM and/or pincer) who also respond to intra-articular block injections.

## Materials and methods

The study was reported in accordance with the Guidelines for Reporting Reliability and Agreement Studies (GRRAS) [30]. The Regional Ethical Review Board in Lund approved the study (Dnr 2014/12), and the participants signed an informed consent form.

### Participants

From 2014 to 2017, all patients referred for non-arthritic hip and groin pain (*n *= 156) to the Department of Orthopedics at Skåne University Hospital in Sweden were consecutively recruited and screened for eligibility. The inclusion criteria were unilateral or bilateral hip/groin pain lasting > 3 months and an age of 18–55 years. The exclusion criteria were as follows: (i) a history of hip surgery; (ii) a hip pathology (i.e., Perthes disease); (iii) verified moderate or severe osteoarthritis (OA) (Tönnis grade > 1); (iv) a history of intra-articular or peri-articular injection with corticosteroids within the last 2 months; (v) palpable hernia; (vi) low-back pain with a positive Lasègue test result with or without an MRI-verified lower back/spine pathology (i.e., spinal stenosis, disc herniation); (vii) other musculoskeletal comorbidities overriding the hip-related symptoms and dysfunction; (viii) comorbidities excluding physical activity and training; (ix) psycho-social disorders; (x) drug abuse; and (xi) an inability to understand the language of interest (any Scandinavian languages or English). Ninety-five patients were eligible, 12 of whom declined participation. Eighty-three participants were consequently recruited. After the initial clinical examination, two participants declined further participation and were thus excluded. Eighty-one participants were finally included in the study (Table 1). The participants rated their perceived pain, disability and associated problems on the Copenhagen Hip and Groin Outcome Score (HAGOS), which includes six subscales: pain, symptoms, physical function in activities of daily living (ADL), physical function in sport and recreation (Sports/rec), participation in physical activity (PA), and quality of life (QOL) (Table 1). The HAGOS has been shown to be a reliable and valid tool in the assessment of long-standing hip and groin pain in a young to middle-aged population [50]. The score for each subscale ranges from 0 to 100, where 0 indicates extreme problems and 100 indicates no problems. The participants rated their preinjury and current activity level on the Hip Sports Activity Scale (HSAS), a valid and reliable questionnaire for assessing individuals’ activity level in this patient group [38] (Table 1).

**Table 1:** Participant characteristics (*n* = 81)

	Mean (SD)
Age (years)	36 (9)
Sex, women (%)	49
BMI (kg/m^2^)	24.8 (3.9)
Unilateral symptoms left/right (*n*)	29/39
Bilateral symptoms (*n*)	13
HSAS preinjury, median* (IQR)	4 (3–6)
HSAS current, median* (IQR)	2 (1–3)
HAGOS scores*	
Symptoms	56.7 (15.4)
Pain	57.7 (17.0)
ADL	62.6 (21.2)
Sport/rec	47.8 (23.3)
PA	29.9 (28.0)
QOL	28.5 (14.6)

Data are expressed as the mean (SD) unless otherwise stated

*HSAS* Hip Sports Activity Scale, *HAGOS* Copenhagen Hip And Groin Outcome Score, *ADL* activities of daily living, *PA* physical activities, *QOL* quality of life

*n* = 72, nine participants did not complete the HAGOS and HSAS questionnaire

### Clinical assessment

The clinical assessment of the hip included clinically relevant hip impingement tests, as described by Martin et al. [32]: the Anterior Impingement Test (AIMT), Flexion/Adduction/Internal Rotation (FADIR) test, Flexion/Abduction/External Rotation (FABER) test, Dynamic External Rotatory Impingement Test (DEXRIT), Dynamic Internal Rotatory Impingement Test (DIRIT), Posterior Rim Impingement Test (PRIMT), and passive ROM (flexion, internal rotation with 90° of hip flexion, internal rotation with a neutral hip position, external rotation with 90° of hip flexion, abduction and extension). All participants were assessed by an orthopedic surgeon (IK). To evaluate the inter-rater agreement, the first 69 participants (hips, *n* = 138) recruited for the study were assessed by both an orthopedic surgeon (IK) and a physical therapist (AP) in a random order. The two raters examined each patient within 1 hour. Both raters had extensive clinical experience in the assessment and treatment of people with hip and groin pain, they examined the patients independently in separate rooms, and they were blinded to the results of the other rater. Prior to the data collection, the raters practiced administering the tests together on two occasions for calibration purposes, and the criteria for a negative/positive test result were agreed upon.

### Hip impingement tests

**Fig. 1:**
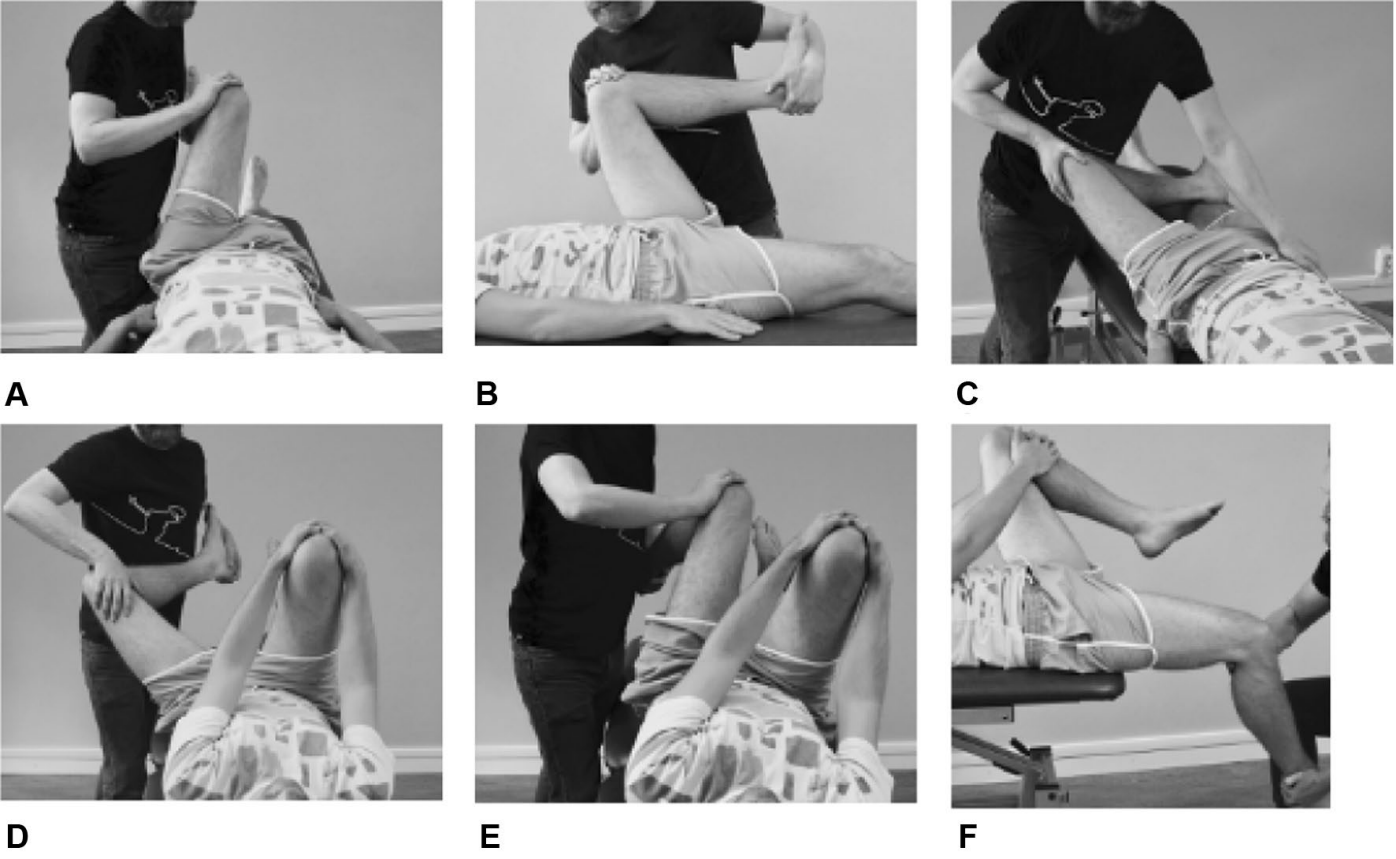
**a**–**f** Hip impingement tests: **a** AIMT: the examiner brings the hip into 90° of flexion and then moves the hip into internal rotation and adduction; **b** FADIR: the examiner brings the hip into maximal flexion, internal rotation and adduction; **c** FABER: the examined leg is placed with the foot just proximal to the contralateral knee joint, the hip is moved into a combined flexion, abduction and external rotation position, and the examiner places a hand on the contralateral side of the pelvis to minimize pelvic rotation; **d** DEXRIT; **e** DIRIT: the patient is asked to hold the contralateral hip in more than 90° of flexion. The examiner then brings the hip into approximately 90° of flexion and moves the hip through a wide arc of extension, abduction and external rotation (DEXRIT) or extension, adduction and internal rotation (DIRIT); and **f** PRIMT: with the patient in the supine position lying at the edge of the examining table, both hips are brought into flexion, and the patient is instructed to keep the contralateral hip in flexion while the examined hip is brought into extension, abduction and external rotation

The six impingement tests were performed according to the methods described by Martin et al. [32] (Fig. 1a–f). All tests were performed in a supine position. The patients were instructed to report any pain in the hip and/or groin area. The test results were categorized as either (1) negative (no pain) or (2) positive (the patient’s typical hip and/or groin pain was reproduced).

### Passive ROM

**Fig. 2:**
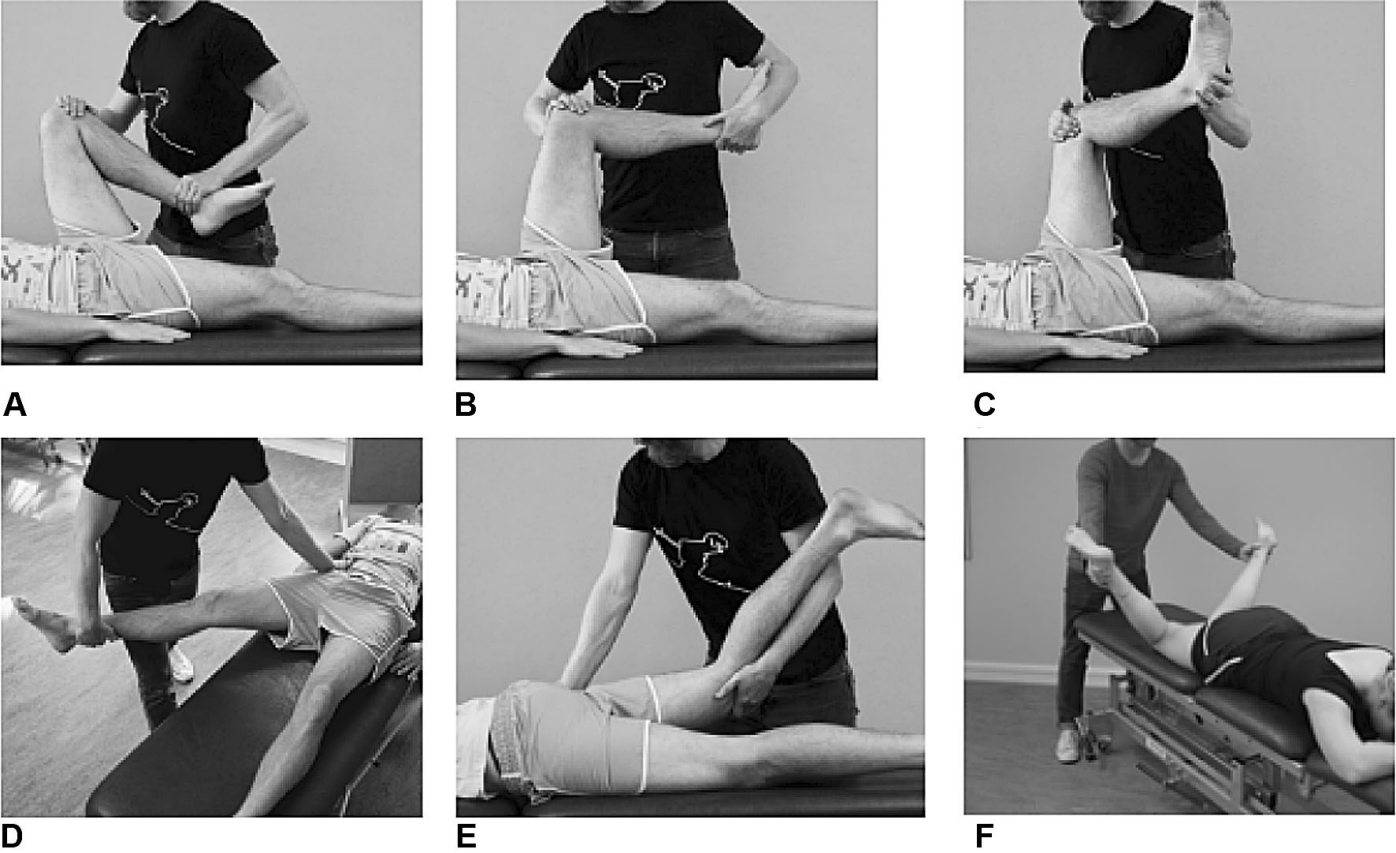
**a**–**f** Passive hip ROM in flexion (**a**), internal rotation with 90° of hip flexion (**b**), external rotation with 90° of hip flexion (**c**), abduction (**d**), extension (**e**) and internal rotation with a neutral hip position (**f**)

Passive flexion (Fig. 2a), internal rotation with 90° of hip flexion (Fig. 2b), external rotation with 90° of hip flexion (Fig. 2c) and abduction (Fig. 2d) were examined with the patient in a supine position. Passive extension (Fig. 2e) and internal rotation with a neutral hip position (Fig. 2f) were examined with the patient in a prone position. The patients were instructed to stay relaxed during the tests and to report any pain experienced in the hip/groin area. Each test result was categorized in a clinical manner as either (1) negative (defined as full ROM with or without pain) or (2) positive (defined as decreased ROM with or without pain).

### Radiographic data

The alpha angle and lateral center–edge angle (LCE angle) were identified and analyzed in accordance with a report by Clohisy et al. [16]. The Lauenstein (frog-leg lateral) projection was used to obtain the alpha angle, whereas the LCE angle was identified on the anteroposterior pelvic view. For seven patients, no Lauenstein projection was available, and the alpha angle was therefore not calculated for these patients. An alpha angle of ≥ 60° was used as the cutoff defining a cam morphology, and an LCE angle of ≥ 40° indicated the presence of a pincer morphology [37]. Excellent inter-rater reliability (ICC ≥ 0.84) for the alpha angle and LCE angle measurements was observed in an analysis of 67 patients from this cohort [40].

### Intra-articular block injection

All injections were performed by the senior orthopedic surgeon (IK) under fluoroscopic guidance. The intra-articular position of the needle was confirmed by the injection of approximately 1 ml of contrast agent iohexol (Omnipaque, 180 mg/ml) prior to a blockage injection of a mixture containing 2 ml triamcinolone (Lederspan, 20 mg/ml), 4 ml ropivacaine (Narop 10 mg/ml) and 4 ml lidocaine (Xylocain 10 mg/ml). The patients were asked to score the severity of pain they experienced on a visual analog scale (VAS), from 0 (no pain) to 100 (maximal pain) mm, prior to the injection and 1, 2, and 4 hours after the injection. During this period, the patients were instructed to perform activities that would normally provoke pain to determine whether there was any improvement in the symptoms [28]. A decrease in the VAS score by 50% or more over a period of 4 h after the injection was considered to indicate a true effect [33]. The patients were categorized as either responders to the injection (≥ 50% decrease in the VAS score) or nonresponders to the injection (< 50% decrease in the VAS score). Seven patients declined the intra-articular injection, and four patients did not complete the VAS scoring after the injection.

### Reference standard

It is recommended to use a triad of symptoms, clinical signs and radiological findings for the diagnosis of FAI syndrome, and to further confirm the hip as the source of pain, an intra-articular block injection can be performed [25]. As the aim of the present study was to evaluate the diagnostic accuracy of the clinical tests, the following criteria for the diagnosis of FAI syndrome had to be fulfilled and were then used as reference standards for evaluating the diagnostic accuracy of the clinical tests: (1) there were symptoms indicating FAI syndrome; (2) there were radiological findings showing CAM and/or pincer morphology; and (3) the patient was considered a responder to the intra-articular block injection.

### Statistical analysis

Cohen’s kappa statistics were used for the inter-rater agreement analysis. The kappa values were interpreted as follows: < 0.00 = poor agreement, 0.00–0.20 = slight agreement, 0.21–0.40 = fair agreement, 0.41–0.60 = moderate agreement, 0.61–0.80 = substantial agreement and 0.81–1.00 = almost perfect agreement [31]. Absolute agreement was expressed in percents. Tests showing kappa values below 0.4 were not included in the analysis of diagnostic accuracy. The results from one rater (the orthopedic surgeon) in the clinical examination were used to determine the diagnostic accuracy. The sensitivity, specificity, positive predictive values (PPVs), and negative predictive values (NPVs) were calculated for each test using cross table analysis [3]. All calculations were performed in SPSS for Windows, V.22.0 (IBM Corp., Armonk, New York, USA). No sample size calculations were performed prior to the study due to its exploratory design. However, the number of participants was selected so that a sample size deemed “good” (*n* = 50–99) was met according to 4-point scale checklist for methodological quality in the consensus-based standards for the selection of health measurement instruments (COSMIN) [48].

## Results

### Inter-rater agreement

The kappa values were greater than 0.6 for the AIMT, FADIR and FABER tests, greater than 0.4 for DEXRIT and DIRIT, and less than 0.4 for the PRIMT. The absolute agreement ranged between 80 and 83% (Table 2). For the passive hip ROM, the kappa values were greater than 0.4 for flexion, internal rotation with 90° of hip flexion, internal rotation with a neutral hip position, external rotation with 90° of hip flexion and abduction, and they were less than 0.4 for extension. The absolute agreement ranged from 78 to 91% (Table 2). Detailed inter-rater agreement data for the two raters are presented in the Appendix.

**Table 2:** Kappa values, 95% confidence intervals (CI 95%) and absolute agreement (%) for the hip impingement tests and the ROM tests (hips, *n* = 138)

Clinical tests of the hip	Kappa value (95% CI)	Absolute agreement (%)
Hip impingement tests		
AIMT	0.665 (0.540; 0.790)	83
FADIR	0.638 (0.509; 0.767)	82
FABER	0.623 (0.498; 0.748)	81
DEXRIT	0.549 (0.402; 0.695)	80
DIRIT	0.561 (0.289; 0.640)	81
PRIMT	0.357 (0.168; 0.546)	80
Passive ROM		
Flexion	0.447 (0.262; 0.632)	82
Internal rotation with 90° hip flexion	0.472 (0.312; 0.632)	78
Internal rotation with neutral hip position	0.431 (0.236; 0.626)	85
External rotation with 90° hip flexion	0.553 (0.346; 0.760)	90
Abduction	0.514 (0.319; 0.709)	87
Extension	0.211 (− 0.07; 0.494)	91

### Diagnostic accuracy

Of the 81 participants, 7 were excluded due to missing radiographs or missing Lauenstein projections, and 11 participants were excluded because they either declined the injection or failed to complete the VAS scoring after the injection. Thus, 63 participants [6 with bilateral symptoms and 57 with unilateral symptoms (hips *n* = 69)] were included in the analysis. The results for the criteria used as reference standards are described in Table 3. The PRIMT and passive extension data were excluded from the analysis of diagnostic accuracy due to poor inter-rater agreement.

**Table 3:** Results from the criteria used as reference standards (hips, *n* = 69)

Criteria for the diagnosis of FAI syndrome	Positive results (hips) *n* (%)
1. Symptoms	69 (100)
2. Radiological findings	46 (67)
3. Responder to intra-articular block injection	48 (70)
All three criteria met	35 (51)

### Hip impingement tests

The AIMT and FADIR test both showed a sensitivity of 80%, whereas the FABER test, DEXRIT and DIRIT had a sensitivity of no higher than 60%. The specificity ranged from 24 to 51% for all five tests. The PPV ranged from 48 to 53%, and the NPV ranged from 45 to 56% for all tests (Table 4).

**Table 4:** The sensitivity, specificity, positive predictive value (PPV) and negative predictive value (NPV) with 95% confidence intervals (CI 95%) for the hip impingement tests and passive hip range of motion (ROM) test (hips, *n* = 69)

Hip impingement tests	Sensitivity % (CI 95%)	Specificity % (CI 95%)	PPV % (CI 95%)	NPV % (CI 95%)
AIMT	80 (67–93)	26 (12–41)	53 (39–66)	56 (32–81)
FADIR	80 (67–93)	24 (9–38)	52 (39–65)	53 (28–79)
FABER	54 (38–71)	38 (22–54)	48 (32–63)	45 (27–63)
DEXRIT	60 (44–76)	46 (29–62)	53 (37–68)	53 (35–71)
DIRIT	54 (38–71)	51 (35–68)	53 (36–69)	53 (36–70)
Passive ROM				
Flexion	51 (35–68)	68 (52–83)	62 (44–79)	58 (42–73)
Internal rotation with 90° hip flexion	56 (39–73)	63 (48–79)	63 (46–79)	57 (42–73)
Internal rotation in neutral hip position	29 (13–44)	94 (86–100)	83 (62–100)	56 (43–69)
External rotation with 90° hip flexion	37 (21–53)	79 (66–93)	65 (44–86)	55 (41–69)
Abduction	46 (29–62)	79 (66–93)	70 (51–88)	59 (44–73)

### Passive hip ROM

The sensitivity for all ROM tests ranged from 29 to 56%. Internal rotation with a neutral hip position showed the highest specificity (94%), followed by abduction and external rotation with 90° of hip flexion (79%), flexion (68%) and internal rotation with 90° of hip flexion (63%). The PPV ranged from 62 to 83%, where the internal rotation with a neutral hip position exhibited the highest PPV value. The NPV ranged from 55 to 59% for all ROM tests (Table 4).

## Discussion

The most important finding of the present study was that the diagnostic accuracy for FAI syndrome can be improved when results from both hip impingement and range of motion tests are considered. The results show substantial agreement between two experienced raters for the AIMT, FADIR and FABER tests, moderate agreement for the DEXRIT and DIRIT, and poor agreement for the PRIMT. Moderate agreement was noted for all hip ROM tests, except the extension test, which showed poor agreement. The AIMT and FADIR test showed high sensitivity but low specificity in the diagnosis of FAI syndrome. The FABER test, DEXRIT and DIRIT showed moderate sensitivity and low to moderate specificity. Low sensitivity but high specificity was noted for ROM values rated as normal or decreased during internal rotation with a neutral hip position, external rotation with 90° of hip flexion, and abduction. Flexion and internal rotation with 90° of hip flexion exhibited moderate sensitivity and specificity.

The AIMT, FADIR, and FABER tests showed substantial agreement between two experienced raters. In line with these findings, Martin et al. reported substantial inter-rater agreement for the FABER test and moderate inter-rater agreement for the FADIR test [34]. Ratzlaff et al. [42] reported absolute agreement values for the AIMT, FADIR, and FABER that were comparable to our findings. However, no kappa values were reported in their study [42], and since absolute agreement values are limited due to the influence of chance and/or prevalent positive and negative outcomes, our results cannot easily be compared to their findings. Based on our results, which are supported by previous findings [34, 42], the results from the AIMT, FADIR and FABER obtained from different raters may reliably be used in clinical practice as well as in research. The moderate agreement observed for the DEXRIT and DIRIT indicates that results obtained from different raters should be interpreted with caution. The PRIMT can be an inappropriate test for this patient group based on the poor inter-rater agreement observed.

ROM values rated as normal or decreased, with or without pain, had at most moderate agreement between raters in the present study. In a previous study, only poor to moderate inter-rater agreement between experienced clinicians was reported for hip ROM in patients with hip OA [15]. In that study [15], hip ROM was visually estimated in degrees, which may be a reason that the inter-rater agreement was weaker in that study than in our study. One reason that moderate or less than moderate inter-rater agreement was observed in the current study may be that pelvic movement [39] and/or involuntary contractile tissue restrictions [12] can affect the assessment used for determining whether the hip ROM is normal or decreased. Thus, the results obtained from different raters indicating normal or decreased hip ROM should be interpreted with caution in both research studies and in the clinic.

None of the tests performed in this study exhibited more than substantial inter-rater agreement. Possible reasons for this result may be participant bias and/or rater bias. A short time span (1 h) between the examinations performed by the raters was chosen to minimize the effect of any day-to-day changes in the patients’ pain and symptoms. In addition, the order of the raters was randomized to reduce the possible effect of increased pain from the first assessment influencing the second assessment. Both raters had extensive clinical experience, and for calibration purposes, they underwent thorough training prior to the study start to gain a clear and common understanding of the tests. However, it is possible that the technique with which the tests were performed varied between the raters.

Tests with high sensitivity have few false negative results and can therefore be used to exclude diagnoses in patients with negative results. The AIMT and FADIR test showed high sensitivity (80%) and may therefore be suitable to exclude FAI syndrome in patients. However, these tests had low specificity and, thus, many false positive results. Many false positive results for impingement tests have also been reported in previous studies [14, 35]. One reason for this result may be that the tests are uncomfortable and/or painful in people without any hip/groin-related pathology/problems and thus do not reproduce the patient’s actual hip/groin pain. In line with our results, a previous systematic review and meta-analysis reported that hip impingement tests, especially the FADIR test, have high sensitivity but low specificity [43]. However, the majority of previous studies investigating the accuracy of clinical tests for the diagnosis of FAI syndrome used diagnostic imaging alone as a reference [4, 5, 7, 20, 51]. Only a few studies used responders to the injection as a diagnostic criterion, but it was not used in combination with imaging criteria [33, 35]. The use of only imaging criteria may compromise the validity of the diagnostic accuracy observed due to the high prevalence (55%) of FAI morphology and labral tears in the asymptomatic population [23]. Although the diagnostic accuracy of intra-articular block injections has not been fully investigated [45], and its value in predicating outcomes after hip arthroscopy has been questioned [6]; it is suggested to be included as an additional diagnostic tool for FAI syndrome [25]. For this reason, we used a combination of symptoms, radiological data and results from diagnostic block injections for the diagnosis of FAI syndrome. However, the diagnostic accuracy for the AIMT and FADIR test did not improve. Thus, based on previous findings [43] and those in the present study, it appears that the impingement tests included in the current study (i.e., the AIMT and FADIR test) can only be used to exclude FAI syndrome.

A restricted hip ROM in patients with FAI syndrome is considered to be a consequence of the bony interaction between the femur and the acetabulum in patients with CAM and/or pincer morphology. In a systematic review, Diamond et al. [19] reported a smaller hip ROM in patients with FAI syndrome than in controls. However, in a more recent systematic review, Freke et al. [24] reported there are no differences in the ROM between patients with FAI syndrome and controls. Additionally, in a large study including more than 400 athletes (approx. 800 hips), no differences in hip ROM were detected between asymptomatic individuals with CAM/pincer morphology and those without such morphology [36]. Thus, it appears to be unclear whether CAM/pincer morphology, with or without symptoms, is associated with a decreased hip ROM. Regardless, a decreased hip ROM, especially in the impingement position (i.e., internal rotation in 90° of hip flexion), is regarded as an important clinical sign for the diagnosis of FAI syndrome [25]. In our study, a restricted ROM during internal rotation with 90° of hip flexion showed only moderate sensitivity and specificity, indicating that the results from this test should be interpreted with caution.

High specificity (94%) was found for hip ROM values rated as either normal or decreased, with or without pain, during internal rotation with a neutral hip position, and substantial specificity (79%) was noted for external rotation with 90° of hip flexion and abduction, indicating that these tests can be used to rule in patients with FAI syndrome when the results are positive. However, the low sensitivity of these tests corresponds to a high number of false negative results. There may be low sensitivity because it is difficult to detect small restrictions in ROM or because a decreased ROM is not always present in patients with FAI syndrome [24]. Additionally, restrictions in the ROM during internal rotation in a neutral hip position and external rotation with 90° of hip flexion might not be caused by bony interactions but rather due to soft tissue restrictions, such as increased capsular thickness [55] and/or involuntary muscle contractions [12]. Restricted hip ROM in all three planes is associated with more severe cases in patients with hip OA [9]. Although it has not been studied, this trend might also be present in patients with FAI syndrome and therefore explain the high specificity of these tests, leading to only severe cases being detected. The use of tests with high specificity and low sensitivity leads to the accurate identification of patients with FAI syndrome when the results are positive, but they will also fail to identify patients with FAI syndrome.

To the best of our knowledge, the current study is the first to investigate the diagnostic accuracy of hip ROM for the diagnosis of FAI syndrome. In a previous study, restricted hip ROM, especially during internal rotation, had high sensitivity in detecting radiographic signs of moderate to severe hip OA [9], and it has been suggested that patients with FAI syndrome are at high risk of early-onset hip OA [1, 2, 8, 21]. Additional studies are needed to confirm our findings, i.e., whether hip ROM tests can be used to rule in patients with FAI syndrome. However, by combining results from hip impingement tests having high sensitivity, i.e., the AIMT and FADIR test, with results from hip ROM tests with high specificity, the diagnostic accuracy of the clinical examination may be improved [44]. Consequently, unnecessary invasive and expensive examinations, such as those involving radiographs, may be limited or avoided.

The main limitation of the study is that all patients were referred to tertiary care and, therefore, likely had a higher pre-test probability of having FAI syndrome than did patients in primary care. Thus, the pre-test probability (51% in our study) is expected to be lower in a primary care cohort. Additional studies of the diagnostic accuracy of clinical tests in a primary care setting are warranted. Another limitation is that only two raters performed the clinical examination, which may limit the generalizability of the results to several raters. Because the clinical tests aim to reproduce the patient’s pain, only two raters were included to limit the amount of discomfort experienced by the patients. A third limitation is that hip ROM was only assessed dichotomously. Additional studies where ROM is measured with a goniometer or inclinometer may provide more insight into possible cut-off values for the hip ROM to identify patients with FAI syndrome. The main strength of the present study is that we used the best available evidence for the diagnosis of FAI syndrome, i.e., a combination of symptoms, radiological findings and patient-reported responses after intra-articular block injections as reference standards to evaluate the diagnostic accuracy of clinical tests. Another strength is that our study is the first to evaluate the diagnostic accuracy of a hip ROM test, not only hip impingement tests.

In the clinical setting, the AIMT and FADIR test may be used to rule out patients when the results are negative. However, if the AIMT or FADIR test results are positive, the evaluation of hip ROM during internal rotation may be used to rule in patients.

## Conclusions

The substantial agreement observed between the two raters for the three impingement tests suggests that the results from different raters are reliable. The moderate or less than moderate agreement for hip ROM implies that results from different raters should be interpreted with caution in both research studies and in the clinic. The AIMT and FADIR test can be used to rule out patients with FAI syndrome when the results are negative, while the evaluation of ROM during internal rotation in a neutral position may be more suitable to rule in patients with FAI syndrome when the results are positive. Both the results of the AIMT and FADIR test and those of a hip ROM test may be used to accurately identify patients who potentially suffer from FAI syndrome.

## Appendix

See Appendix Tables

**Table 5:** Crosstab of inter-rater agreement data for rater A and rater B for the impingement tests

Impingement tests		Rater A	
	Rater B	Negative	Positive
AIMT	Negative	63	12
	Positive	11	52
		Negative	Positive
FADIR test	Negative	55	14
	Positive	11	58
		Negative	Positive
FABER test	Negative	66	3
	Positive	23	46
		Negative	Positive
DEXRIT	Negative	77	12
	Positive	16	33
		Negative	Positive
DIRIT	Negative	82	12
	Positive	14	30
		Negative	Positive
PRIMT	Negative	99	8
	Positive	19	12

^5^,

**Table 6:** Crosstab of inter-rater agreement data for rater A and rater B for the ROM tests

Passive hip ROM		Rater A	
	Rater B	Negative	Positive
Flexion	Negative	99	12
	Positive	12	15
		Negative	Positive
Internal rotation with 90° hip flexion	Negative	83	13
	Positive	17	25
		Negative	Positive
Internal rotation with neutral hip position	Negative	106	4
	Positive	17	11
		Negative	Positive
External rotation with 90° hip flexion	Negative	113	8
	Positive	6	11
		Negative	Positive
Abduction	Negative	107	10
	Positive	8	13
		Negative	Positive
Extension	Negative	124	3
	Positive	9	2

^6^.
